# Shear Bond Strength of Four Types of Orthodontic Retainers after Thermocycling and Cyclic Loading

**DOI:** 10.1155/2021/9424040

**Published:** 2021-06-30

**Authors:** A. Golshah, Z. Amiri Simkooei

**Affiliations:** ^1^Department of Orthodontics, Faculty of Dentistry, Kermanshah University of Medical Sciences, Kermanshah 6715847141, Iran; ^2^Students Research Committee, Faculty of Dentistry, Kermanshah University of Medical Sciences, Kermanshah 6715847141, Iran

## Abstract

**Objectives:**

This study assessed the shear bond strength (SBS) of four types of orthodontic retainers after thermocycling and cyclic loading.

**Materials and Methods:**

This in vitro, experimental study evaluated 120 extracted mandibular central and lateral incisors. The teeth were mounted in acrylic resin blocks in sets of three, such that the interdental contacts and positioning of the teeth resembled the dental arch. The acrylic blocks were divided into four groups (*n* = 10) for the use of 0.016 × 0.022-inch Bond-A-Braid® wire, 0.0195-inch twisted wire, 0.0175-inch coaxial wire, and 0.038 × 0.016-inch Ortho-Flex Tech® wire, as retainers. The retainers were bonded to the lingual surface of the teeth with Transbond XT adhesive in all groups, and the specimens underwent thermocycling and cyclic loading (125,000 load cycles applied to the incisal edge of the incisor tooth in the middle, simulating 6 months of clinical service). Any fracture in the process of aging was recorded. The teeth were then subjected to vertical loads applied along their occlusoapical axis in a universal testing machine to determine the SBS in Newtons. The adhesive remnant index (ARI) scores were also determined. Data were analyzed using ANOVA, the Monte Carlo chi-square test, and the Kruskal–Wallis test.

**Results:**

Thermocycling and cyclic loading did not cause degradation or fracture of the retainers. The SBS and ARI scores of the four groups were not significantly different (*P* > 0.05).

**Conclusion:**

The SBS of retainers with flat rectangular-shaped cross-section was similar to that of retainers with a round cross-section; thus, they have no superiority over each other in this respect.

## 1. Introduction

Maintaining the orthodontic treatment results after the completion of treatment is highly important [1]. Retention is mandatory following orthodontic treatment to prevent relapse. Relapse is an unpredictable phenomenon which is variable in different individuals [2]. Several factors are involved in the occurrence of relapse following completion of orthodontic treatment such as the abnormal function of the muscles, occlusal stresses, and regeneration of periodontal fibers [3]. Also, by a reduction in the length of dental arch over time, crowding of the anterior teeth increases [4]. Thus, it appears that the use of fixed lingual retainers is the only way to maintain the ideal alignment of the teeth following completion of orthodontic treatment [5, 6]. Long-term studies have confirmed that lingual retainers can effectively maintain the new position of mandibular incisors following orthodontic treatment. Use of lingual retainers is even more important when the intercanine width needs to be maintained after treatment and also when the supporting periodontal tissue is lost [1].

A series of orthodontic wires are used as retainers. They are attached to the lingual surface of the maxillary and particularly mandibular teeth. Despite the available reports regarding the acceptable survival of lingual retainers, fracture of the retainers and their adhesive debonding from the tooth surface are still among the most common types of clinical failures [1]. The material and structure of the retainer, type of composite resin used for bonding of the retainer, and the position of the retainer (maxilla or mandible) are among the most influential factors affecting the survival and success of lingual retainers [7]. Fracture at the wire-composite interface, wire fracture due to stress accumulation at the bending points, and detachment of resin pads present at the enamel-composite interface are among the main problems encountered in the use of lingual retainers. According to the available in vitro studies on bond strength, detachment of a splinted wire is commonly a cohesive type of failure, taking place at the interface of the wire and composite [8, 9]. Failure at the wire-composite interface is attributed to two main factors: toothbrushing and mastication that often result in thinning and weakening of the resin pad, and propagation of internal cracks due to movement of the retainer between the overlying and underlying resin pads in the process of physiological tooth movement [7].

Different types of wires have been used for assessment of the bond strength of retainers such as flat-braided wire (Bond-A-Braid®, Reliance Orthodontic Products), three-strand wire (Ortho Technology) [1], five-strand wire (PentaOne, Masel), dead-soft eight-braided wire (Bond-A-Braid, Reliance), dead-soft coaxial wire (Respond, Ormco) [4], fiber-reinforced composite (InFibra Ribbon, Italy) [2], polyethylene ribbon reinforced, and braided stainless steel wire [7].

Some previous studies reported that the debonding forces were not significantly different for different types of retainers [1, 4, 7]; while, some others reported significant differences in debonding forces of different retainers [2, 10]. Considering this controversy and gap of information regarding the rectangular multibraided ribbon arc wire, this study aimed to assess the shear bond strength (SBS) of four types of bonded retainers and their adhesive remnant index (ARI) score. The tested null hypothesis was that the SBS of Bond-A-Braid and Ortho-Flex wires with rectangular-shaped cross-section and the larger contact area with the lingual surface of the tooth would not be significantly different from the SBS of 0.0175 and 0.0195-inch multistranded wires with a round cross-section.

## 2. Materials and Methods

### 2.1. Study Samples

This in vitro, experimental study evaluated 120 mandibular central and lateral incisors extracted due to poor periodontal prognosis. The study protocol was approved by the Ethics Committee of Kermanshah University of Medical Sciences (IR.KUMS.REC.1399.837).

#### 2.1.1. Sample Size Calculation

The minimum sample size was calculated to be 10 in each group (a total of 40) according to a study by El-Sorogy et al. [2], assuming the standard deviation of the debonding force to be 12.28 N in the Bond-A-Braid group and 15.73 N in the FRC group, *d* = 21, *α* = 0.05, and study power of 90%.

#### 2.1.2. Inclusion Criteria

The inclusion criteria were the absence of cracks or defects on visual inspection of the teeth and sound lingual surface of the teeth with no restoration or caries.

#### 2.1.3. Intervention

Immediately after extraction, the teeth were thoroughly rinsed with water, and the residual soft tissue and calculus were removed by a scaler. The teeth were stored in distilled water at room temperature to remain hydrated [2]. The teeth were cleaned with slurry water and a prophy brush prior to mounting in acrylic blocks. Next, they were mounted in resin blocks in sets of three, such that the interdental contacts and the position of the teeth simulated the dental arch. The mesiodistal width of the middle tooth in all blocks was almost the same (5 mm). For this purpose, the mesiodistal width of all teeth was measured, and those with the same dimensions were selected. Next, the roots were dipped in melted wax to 2 mm below their cementoenamel junction, such that the roots were coated with one layer of wax with 0.5–1 mm thickness. The teeth were subsequently mounted in autopolymerizing acrylic resin, and after completion of polymerization of the acrylic resin, the acrylic blocks were placed in boiling water to eliminate the wax layer covering the roots. The teeth were removed from the blocks, and light-body elastomeric impression material was placed in the acrylic block. The teeth were placed back in the acrylic resin, and excess material was removed. This was performed to simulate the periodontal ligament and physiological mobility of the teeth [7]. The acrylic blocks were then randomly divided into four groups (*n* = 10):

  Group 1: 0.016 × 0.022-inch wire (Bond-A-Braid®, Reliance Orthodontic Products, Itasca, IL, USA)
  Group 2: 0.0195-inch twisted wire (Ortho Technology, Tampa, Florida, USA)
  Group 3: 0.0175-inch coaxial wire (Ortho Technology, Tampa, Florida, USA)
  Group 4: 0.038 × 0.016-inch wire (Ortho-Flex Tech®, Reliance Orthodontic Products, Itasca, IL 60143, USA)

**Figure 1:**
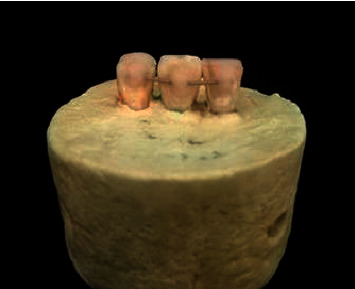
Mounting of the teeth in sets of three.

Next, for bonding of the retainers, the lingual surface of the teeth was polished with pumice, rinsed, air-dried, and etched with 37% phosphoric acid for 30 s. The surface was then thoroughly rinsed and dried with air spray for 20 s. The etched surface had a chalky white appearance after drying. Transbond XT adhesive (#M Unitek, Monrovia, CA, USA) was applied on the etched surface and cured for 40 s. Next, 10 mm of the passive retainer wire was bonded to the lingual surface of the teeth parallel to the acrylic base using Transbond XT adhesive (Figure 1). The same type of adhesive (Transbond XT) was used in all four groups. Light-curing was performed using a LED curing unit (LED H Ortho, Guilin Woodpecker Medical Instrument Co., China) with a light intensity of 1800 mW/cm^2^ from all directions for 40 s. To ensure adequate polymerization, the tip of the curing unit had 2 mm distance from the resin surface [2]. The amount of composite used was standardized by using a minidome-shaped Mold™ (Ortho-Care Ltd., Bradford, West Yorkshire, UK).

#### 2.1.4. Aging

**Figure 2:**
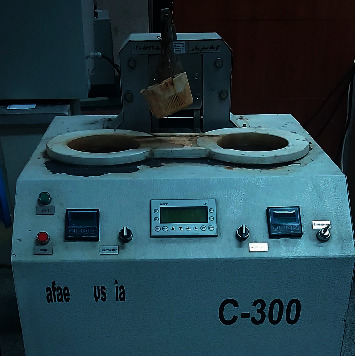
Thermocycler.

**Figure 3:**
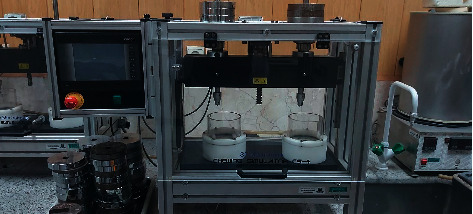
Cyclic loading machine.

The specimens then underwent thermocycling with 10,000 thermal cycles (Figure 2). Next, they were mounted in the cyclic loading machine and subjected to 125,000 load cycles applied to the incisal edge of the incisor tooth positioned in the middle to simulate the masticatory forces applied to the teeth during a 6-month period in the clinical setting (Figure 3) [2]. Any fracture in the aging process was recorded.

#### 2.1.5. Shear Bond Strength Test

**Figure 4:**
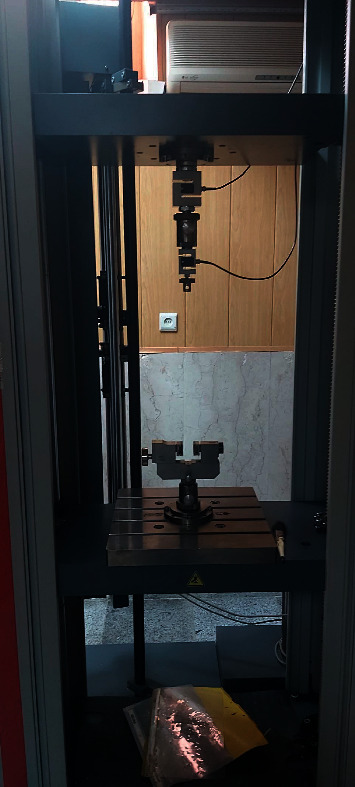
Universal testing machine.

The specimens were then transferred to a universal testing machine (Z020, Zwick/Roell, Ulm, Germany). The clamp of the machine was positioned at the middle of the wire, and vertical load was applied to the teeth along their occlusoapical axis at a crosshead speed of 1 mm/min to simulate the bite force (Figure 4). The load applied to the wire was gradually increased until debonding occurred, and the SBS was recorded in Newtons (N).

#### 2.1.6. Adhesive Remnant Index

**Figure 5:**
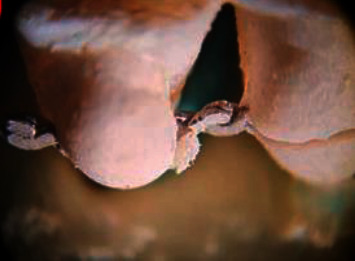
ARI score 3 fracture.

The ARI scores were then determined by quantifying the amount of adhesive remaining on the enamel surface of each tooth where the debonding occurred according to Artun and Bergland [11]. For this purpose, the teeth were inspected under a stereomicroscope (Leica 245E, USA) at x20 magnification [1, 2]. The ARI scores were determined as follows [12]:

  Score 0: no adhesive remaining on the enamel surface
  Score 1: <50% adhesive remaining on the enamel surface
  Score 2: >50% adhesive remaining on the enamel surface
  Score 3: the entire adhesive remaining on the enamel surface (Figure 5)

### 2.2. Statistical Analysis

The Kolmogorov–Smirnov test was used to analyze the normality of data distribution. Since the SBS data had normal distribution (*P* > 0.05), ANOVA was applied for general comparison of SBS of the groups. The Monte Carlo chi-square test was applied for the comparison of the frequency of ARI scores among the groups. The Kruskal–Wallis test was used for the comparison of the median ARI score. All statistical analyses were performed using SPSS version 18 (SPSS Inc., IL, USA) at 0.05 level of significance.

## 3. Results

**Figure 6:**
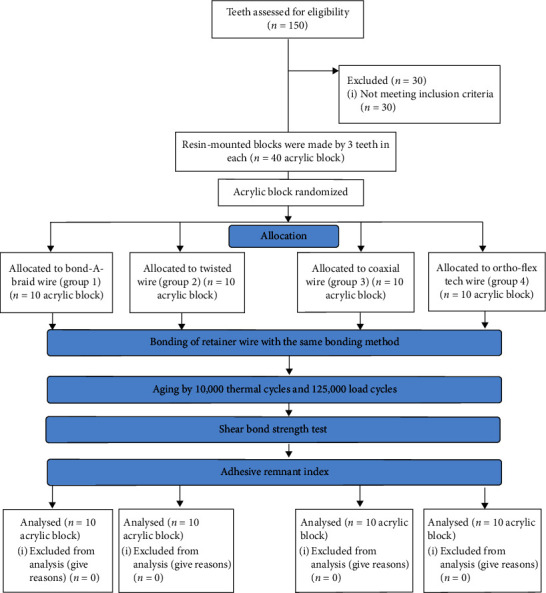
Flow diagram of the study.

Figure 6 shows the flow diagram of the study.  Table 1 presents the mean SBS of the four groups. ANOVA revealed no significant difference in SBS of the four groups (*P*=0.239).

Table 2 presents the ARI scores of the four groups. The Monte Carlo chi-square test revealed no significant difference in the frequency distribution of ARI scores among the four groups (*P*=0.738). The Kruskal–Wallis test revealed no significant difference in the median ARI score between the four groups either (*P*=0.239).

## 4. Discussion

Relapse following orthodontic treatment is a common postoperative complication. Thus, the long-term use of fixed retainers is often recommended to maintain the treatment results [2]. Fixed retainers not only prevent postoperative changes but also prevent the crowding caused by late mandibular growth [13]. A previous study showed that patients using fixed lingual retainers had a superior dental alignment than those not using aligners, after a 5–10-year period. Fixed retainers have no harmful effects on the oral hard or soft tissue [4]. However, occlusal changes have been observed in patients with long-term fixed retention, such as unexpected torque changes between the adjacent teeth or opposite inclinations of contralateral mandibular canines [14]. In extreme situations, destruction of the buccal alveolar bone and occurrence of gingival recession have been observed [15–17]. Failure of retainers more commonly occurs within the first 12 months [18]. Hence, it is important to study the alterations of aged retainers since the incisors are more susceptible to relapse after orthodontic treatment [19].

This study evaluated four different types of retainer wires, namely, Band-A-Braid, Ortho-Flex, and 0.0175-inch and 0.0195-inch multistrand wires, which were all bonded with one type of adhesive (Transbond XT). A previous study concluded that the retainer wire selection was more important than the composite selection [20].

In the oral cavity, lingual retainers undergo cyclic stresses due to mastication, occlusion, and parafunctional habits [21, 22]. Repetition of subcritical loading induces fatigue and may lead to total or partial fracture of one or more components of the retainer complex. Although such forces are often below the in vitro maximum debonding threshold, they may have the same destructive effect as high-magnitude sudden impacts that rarely take place in the clinical setting [21, 22]. Therefore, it is expected that fatigue tests clarify the clinical durability more accurately than the static tests [21, 22]. However, prediction of the degree of fatigue required to induce failure in initially sound specimens may not be easily. Thermocycling and cyclic loading are commonly performed in vitro to simulate the intraoral loads. Moreover, vertical loads are applied to the specimens to assess their strength and resistance.

The SBS of retainers must be high enough to resist masticatory stresses. Bond failure increases the chair time and the costs and is inconvenient for patients [23]. This study assessed the SBS of four types of wires commonly used as fixed lingual retainers in the clinical setting. The tested null hypothesis was that the SBS of Band-A-Braid and Ortho-Flex wires with rectangular-shaped cross-section and larger contact area with the lingual surface of the teeth would not be significantly different from the SBS of 0.0175-inch and 0.0195-inch multistrand wires with a round cross-section. In this study, the teeth underwent 10,000 thermal cycles and 125,000 load cycles with 20 N load to simulate 6 months of clinical service [2]. None of the specimens were damaged in this process. The results showed no significant difference in SBS of the four groups; thus, the null hypothesis of the study was accepted. Similarly, Cook et al. [1] concluded that the use of flat-braided wires is not as widely reported as circular cross-sectional wires in relation to bonded retainers. The SBS of 0.0195-inch three-strand wire was around 16 N higher than that of 0.0175-inch six-strand wire in the present study, although it was not significant. It may be assumed that higher number of strands in the retainer wire does not increase the bond strength; however, future clinical studies are required to confirm this statement. The current results were in agreement with the findings of some and in contrast to the results of some others. The results of Samson et al. [24] were in line with the present findings, since they found no significant difference in the debonding force of round and flat wires, and the debonding force of Bond-A-Braid wire in their study (56.63 N) was almost similar to the value obtained in the present study (55.57 N). Cooke et al. [1] measured the debonding force of flat and round Bond-A-Braid wire and 0.0175-inch three-strand wire and reported no significant difference. However, the debonding force of each wire was lower than the corresponding value in the present study, which may be due to different methodologies. They mounted two teeth in each block and had only one site of load application. Moreover, the teeth did not undergo thermocycling or cyclic loading prior to bond strength testing in their study. Aldree et al. [25] assessed the debonding force of flat Ortho-Flex and 0.0215-inch six-strand round retainers and reported a higher debonding force for 0.0215-inch six-strand round wire. The debonding force of flat Ortho-Flex wire in their study was close to the value obtained in the present study; although, their study was conducted on premolar teeth with a highly different morphology compared with mandibular incisors. Kotta et al. [10] measured the debonding force of three types of retainers and reported a significant difference between them. However, the debonding force of Bond-A-Braid flat wire (56.11 N) was close to the value obtained in the present study. They mounted the teeth in sets of two in their study. Also, they did not perform thermocycling or cyclic loading prior to measuring the SBS of specimens. These factors may explain the difference between their results and ours.

The ARI scores were also assessed in the present study, which are important in determining the location of debonding. The results showed no significant difference in the frequency of ARI scores between the groups, which was in line with the results of Scribante et al. [26], El-Sorogy et al. [2], Cook et al. [1], and Kotta et al. [10] but different from the results of Foek et al. [7]. In the latter study, the mode of failure was the same for all specimens. However, the majority of failures showed ARI score 1 in Interlig and DentaPreg retainers. ARI score 2 was noted in 80% of failures in everStick Ortho retainers. The ARI scores were variable in the other two retainers. In the present study, the frequency of ARI scores 2 and 3 was higher than ARI score 0. In the studies by Cook et al. [1] and Milheiro et al. [12], ARI score 3, i.e., debonding at the interface of the retainer and adhesive was the dominant mode of failure. However, in the study by Radlanski and Zain [27], ARI score 0 was the dominant type, i.e., debonding at the interface of adhesive and enamel. ARI score 0 may be due to enamel surface contamination, poor moisture control and isolation, underetching or overetching of the enamel, inadequate drying, or incorrect bonding process.

Reynolds et al. [28] and Reicheneder et al. [29] found that a vertical thrust yielded the highest SBS compared with a tensile force in horizontal or vertical direction. However, SBS depends on both direction and location of the applied force. Several protocols are used to measure the SBS of retainers. However, due to the lack of standardization, their scientific comparison is difficult and inaccurate. Most previous investigations applied a vertical load directly to the bonding interface of orthodontic attachment, and only limited studies, including the present study, applied load to the middle of the interdental part of the wire [2]. Radlanski and Zain [27] demonstrated that the load required for debonding is lower when it is applied to an area other than the bonding interface, compared with direct load application to the interface. Thus, we applied load to the interdental part of the wire, which was a strength of this study. Another advantage of this study, compared with previous investigations, was evaluation of teeth in sets of three; while, most previous studies used single specimens or teeth in sets of two. Since there were two interdental areas for load application in the present study, we used a two-headed clamp to apply vertical load to the interdental part of the wire. Also, one layer of elastomeric impression material was used over the root surface to simulate the periodontal ligament and physiological tooth mobility, which was another strength of this study.

One limitation of in vitro studies, such as the present investigation, is difficult simulation of the loads applied to the teeth in the clinical setting. Accordingly, Cooke et al. [1] discussed that by vertical application of load, a combination of tensile, shear, and torsional forces would be probably applied to the teeth. Also, factors such as age of the enamel, degree of enamel mineralization, lingual surface morphology, and size of teeth affect the magnitude of load required for debonding [1]. Moreover, the final success of bonded retainers is determined by the size of teeth and the quality of occlusal forces applied to them [1]. Teeth with larger crowns have a larger bonding area, which would result in load distribution in a wider enamel surface. Although this was a limitation of our study, we used a mold to standardize the composite volume used for bonding of retainers in all teeth. Thus, the bonding surface area was the same in all teeth.

In vitro studies have some limitations in complete simulation of the clinical environment; thus, their results cannot be directly generalized to the clinical setting. Future clinical studies are required to obtain more reliable results.

## 5. Conclusion

The SBS of retainers with flat rectangular-shaped cross-section was similar to that of retainers with a round cross-section; thus, they have no superiority over each other in this respect.

## Figures and Tables

**Table 1:** Mean SBS (*N*) of the four groups (*n* = 10).

Wire	Mean (*N*)	Std. deviation	*P* value^*∗*^
Bond-A-Braid	55.57	19.16	0.239
Twisted wire	72.08	24.40	
Coaxial	55.92	23.13	
Ortho-Flex Tech	64.88	14.51	

^*∗*^ANOVA.

**Table 2:** ARI scores in the four groups.

ARI score	Bond-A-Braid		Twisted wire		Coaxial		Ortho-Flex Tech		*P* value
	Count	%	Count	%	Count	%	Count	%	
Score 0	1	10.0	0	0.0	3	30.0	2	20.0	0.738^†^
Score 1	1	10.0	1	10.0	1	10.0	0	0.0	
Score 2	4	40.0	5	50.0	5	50.0	5	50.0	
Score 3	4	40.0	4	40.0	1	10.0	3	30.0	
Minimum-maximum	0–3		1–3		0–3		0–3		0.239^‡^

^†^Monte Carlo chi-square test. ^‡^Kruskal–Wallis test.

## Data Availability

The data used to support the findings of this study are available from the corresponding author upon request.
